# Vaccination and transfusion safety at the time of expanding use of live attenuated vaccines

**DOI:** 10.1111/vox.70272

**Published:** 2026-04-20

**Authors:** Pierre Tiberghien, Pierre Gallian, Virginie de La Taille, Caroline Bacquet, Syria Laperche

**Affiliations:** ^1^ Etablissement Français du Sang La Plaine St‐Denis France; ^2^ UMR RIGHT 1098, Université Marie et Louis Pasteur, INSERM, Etablissement Français du Sang Besançon France

Vaccination is among the most powerful public‐health interventions in human history, preventing millions of deaths each year [1]. Yet the success of vaccination programmes has implications for transfusion safety. Vaccines, notably new vaccines, prompt the same practical questions for blood establishments: when is it safe for recently vaccinated individuals to donate blood? How to effectively prevent blood donation by a vaccinated donor when deemed unsafe for the recipient?

These questions—often regarded as a routine operational detail—have regained relevance as the landscape of live attenuated vaccines expands, from increasingly administered ‘old’ vaccines such those for yellow fever and measles–mumps–rubella (MMR) to the introduction of new vaccines such as those for dengue and chikungunya (Figure 1). Live attenuated vaccines will replicate briefly within the host to elicit robust and durable immunity [2]. In most healthy individuals, this replication is well controlled. However, in immunodeficient persons—whether due to congenital defects, malignant haemopathies or immunosuppressive therapies in the context of transplantation, cancer or auto‐immune diseases—the attenuated virus may persist and possibly cause severe disease or fatality [3]. These observations not only justify vaccination contraindications or careful risk–benefit assessments in such populations but also raise a transfusion‐safety concern: an attenuated vaccine strain present in donated blood could cause harm to immunocompromised transfusion recipients, even if it is clinically silent in the donor. Protecting this highly vulnerable group is therefore central to the rationale for post‐vaccination donor deferral. Standard precautionary deferral periods for live attenuated vaccines (typically 2 weeks in the United States and 4 weeks in Europe and per World Health Organisation (WHO) guidance [4]) were established decades ago, largely on theoretical grounds.

**FIGURE 1:**
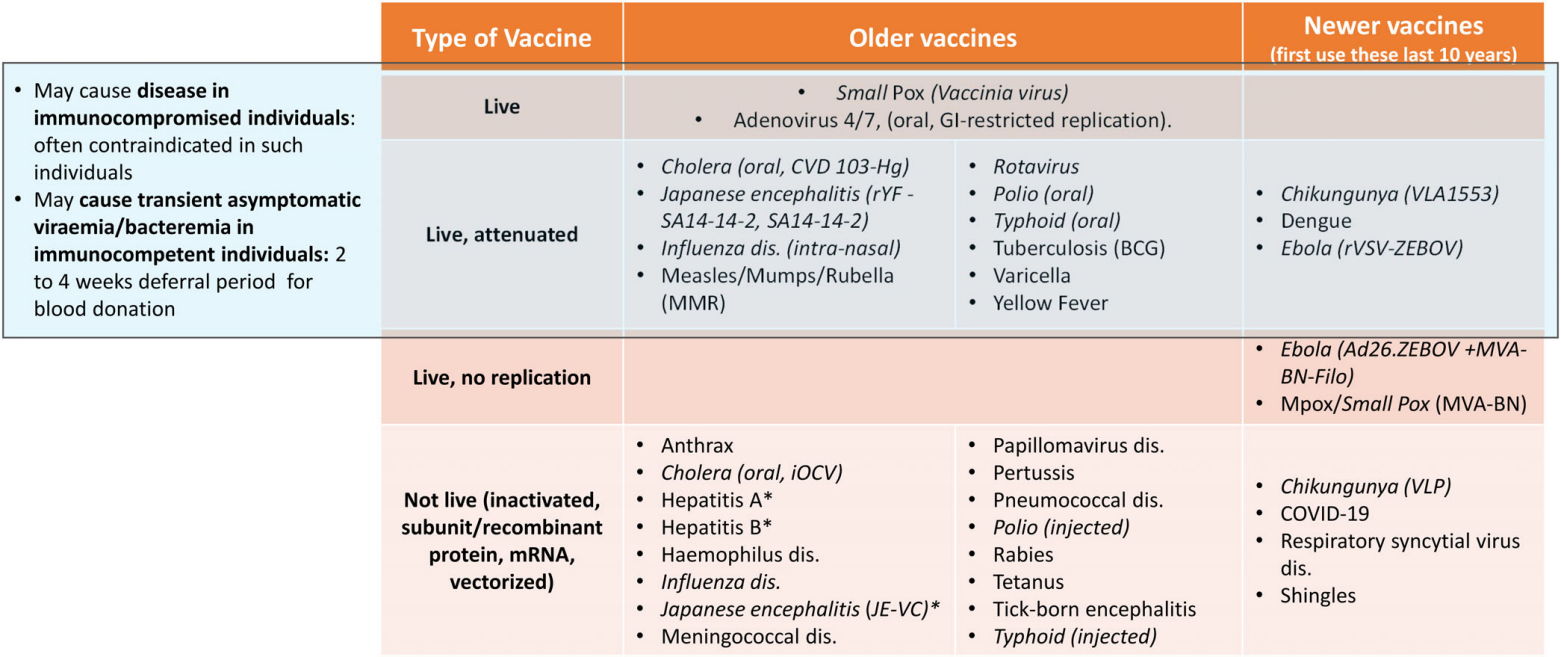
Older and newer vaccines classified according to their main characteristics. Diseases for which vaccines of differing characteristics are available are indicated in italics (with vaccine specifications). *Short period deferral may be applied to prevent spurious detection of non‐infectious vaccine material early on. Ad26.ZEBOV, adenovirus serotype 26‐Zaire Ebola virus; BCG, Bacillus Calmette‐Guerin; GI, gastro‐intestinal; iOCV, inactivated oral cholera virus; JE‐VC, Japanese encephalitis‐vero cell; MVA‐BN‐Filo, modified vaccinia Ankara (Bavarian Nordic)‐filovirus; rVSV‐ZEBOV, recombinant vesicular stomatitis virus—Zaire Ebola virus; rYF, recombinant yellow fever; VLA, developer code; VLP, virus‐like particle.

## USE OF LIVE ATTENUATED VACCINES IN ADULTS

Historically, live attenuated vaccines were administered mostly in childhood, with adult use limited to specific situations such as travel or outbreak control. Classic live vaccines such as MMR were largely confined to childhood programmes or occasional booster campaigns. Consequently, the overlap between recently vaccinated individuals and the adult blood donor population was very limited.

This picture is changing. Yellow fever outbreaks have increased in recent years in South America and Africa. Global mobility has expanded adult indications for travel‐related vaccines such as yellow fever. Furthermore, new, live attenuated vaccines are being developed and licensed specifically for adults, altering the exposure landscape in individuals of an age eligible for blood donation. Lastly, recent measles outbreaks linked to decreasing vaccine coverage have prompted calls for adults to check their vaccination status [5].

For dengue, the live attenuated tetravalent vaccine TAK‐003 (*Qdenga*) is recommended in several endemic countries for children and adolescents [6]; yet, national programmes and occupational risk contexts are already exploring extension to adult cohorts. For chikungunya, a live attenuated vaccine VLA1553 (*Ixchiq*) is now available [7], although not widely. While currently deployed in endemic regions, such vaccines are increasingly considered for immunization of traveling adults from non‐endemic regions.

Lastly, emerging trends in vaccination practices, particularly in the United States, appear to be associated with delays in routine immunization schedules [8], potentially resulting in traditional childhood vaccines being administered later in life.

These evolutions have direct implications regarding donor selection practices. Blood donors—previously a population with little exposure to live attenuated vaccines—are now increasingly likely to be vaccinated with such vaccines. Even though viraemia is brief and transmission risk remains extremely low, the rising proportion of adult vaccines may increase the probability for a blood donor to have been recently vaccinated.

## LESSONS FROM EXPERIENCE: RARE BUT INFORMATIVE EVENTS

Two well‐documented transfusion‐mediated transmissions of live attenuated vaccine strains with varying clinical consequences have been reported. Both involve yellow fever vaccine. In a first report, six military trainees vaccinated with yellow fever vaccine 6 days before blood donation contributed to components subsequently transfused to five recipients. No clinical illness occurred, but serological conversion consistent with exposure was demonstrated [9]. The second report was more dramatic and highlights potential transmission chains that can link vaccination, transfusion and transplantation. In 2023, four solid‐organ recipients developed encephalitis 2–6 weeks after transplantation from a common organ donor who had very recently been transfused [10]. Investigation revealed that one of the involved blood donors had received the yellow fever vaccine 6 days before donating. Yellow fever was confirmed in all four recipients—vaccine‐strain RNA was identified in the brain tissue of one—and two recipients died. Donor deferral according to current practice for yellow fever vaccine would have most probably prevented these transmission cases.

Reported transfusion‐transmitted vaccine infections are exceedingly rare, considering an estimated 280 million blood components are transfused yearly worldwide [11]. However, underreporting, as well as undetected cases prevent a reliable estimate of their true incidence.

Furthermore, transplantation offers cautionary parallels. A transfusion‐related West Nile viraemia in an organ donor resulted in high morbidity and mortality in several transplant recipients [12]. A large majority of organ donors receive transfusions shortly before organ recovery [13], thus creating extensive chains of potential donor‐mediated transmission. Furthermore, organ transplant recipients are frequently transfused at the time of or early after transplantation [14], concurrently to high‐intensity immunosuppression. Overall, these findings demonstrate how low‐probability events can have amplified consequences in such a setting.

## DONOR DEFERRAL IN PRACTICE: HOW EFFECTIVE ARE WE?

In France, donor deferral rules for vaccination follow national guidelines derived from the European Directive 2004/33/EC and WHO recommendations: deferral for 4 weeks after live attenuated vaccines and no deferral for inactivated products (with the exception of a brief deferral after hepatitis A and B vaccination to prevent the spurious detection of non‐infectious vaccine material early on).

Internal Etablissement Français du Sang (EFS) data show that the system functions efficiently but not perfectly. In 2022, 2123 donors (<0.1% of all donations) were properly deferred because of recent vaccination, rising slightly in 2023 and 2024. A much smaller number notified vaccination only after donation (15 in 2024)—early enough in most cases to interdict components, but too late in six instances since 2017, including MMR vaccination. No adverse events were reported in any recipients.

These figures demonstrate both the rarity of such events and the continued need for systematic vigilance and education. They also highlight the limitations of self‐declaration: donor recall and exact knowledge of recent vaccinations are imperfect, and donation staff may not always recognize newly marketed live attenuated vaccines.

## BIOLOGICAL NUANCES: NOT ALL BLOOD DONORS ARE EQUAL

Beyond procedural compliance, emerging immunological findings suggest that *host factors* can modulate vaccine viraemia kinetics in individuals presumed healthy. Individuals with neutralizing anti‐type 1 interferon auto‐antibodies or with inborn error of interferon pathways can develop highly deleterious uncontrolled replication of live attenuated vaccine strains [15, 16]. Our recent finding that live vaccine chikungunya virus can be detected in plasma more than 30 days after *Ixchiq* vaccination in a healthy blood donor [17] further highlights the existence of unexpected inter‐individual variability in viral clearance, even in individuals without overt immunodeficiency. Assessing more thoroughly the reality of ‘immunocompetent’ blood donors in relation to the length of post‐vaccination deferral periods may be timely.

## DIGITAL INTEGRATION, EVOLVING TECHNOLOGIES, POLICY CONSIDERATIONS AND FURTHER DIRECTIONS

Linking health record registries with blood donor databases could significantly improve donor‐selection accuracy while contributing to recipient and donor health. Automatic alerts for recent live vaccine administrations would prevent inadvertent donations and reduce reliance on manual self‐reporting. Such linkage is feasible. Barriers are regulatory and economic.

Pathogen‐reduction technologies applied to blood components may also provide an additional safety layer, while recognizing that such technologies do not currently apply to red blood cell components, remain expensive, may affect blood component quality, and may not be equally effective against all live vaccine strains.

Vaccination‐related deferral periods should reflect ‘best science’ data on vaccine‐viraemia duration and vaccine‐virus properties rather than traditional categories alone. New or experimental vaccines must be rapidly incorporated into donor questionnaires, ideally through centralized electronic systems shared across blood establishments. Close communication between vaccination programmes, public health agencies and blood services can ensure timely information flow—for example, advance notice of mass vaccination campaigns targeting populations that overlap with regular donors.

## CONCLUSION

Vaccines remain a triumph of modern medicine—but an operational challenge for transfusion services. The exceedingly rare but well‐documented cases of transfusion‐mediated vaccine virus infections in transfusion recipients, coupled with the expanding use of live vaccines in adults, invite renewed attention on donor deferral policies and practices.

When transfusion‐mediated vaccine virus infections occur, their consequences may be devastating not only for the recipient but also for the donor. The discovery that a gesture of pure altruism may have transmitted harm can be profoundly distressing. This emotional and ethical dimension reminds us that transfusion safety should protect both recipients and donors. By combining evidence‐based deferral intervals, enhanced digital integration and ongoing scientific vigilance, we can ensure that the progress achieved through vaccination continues to align with the safety expectations of transfusion medicine.

## AUTHOR CONTRIBUTIONS

P.T. wrote the first draft of the manuscript. P.G., V.d.L.T., C.B. and S.L. contributed the data and reviewed the manuscript. Language editing of parts of the manuscript was assisted by ChatGPT (OpenAI).

## CONFLICT OF INTEREST STATEMENT

All authors are employed by Etablissement Français du Sang, the French transfusion public service in charge of collecting, processing, testing and issuing blood components in France.

## Data Availability

Data sharing not applicable to this article as no datasets were generated or analysed during the current study.
